# Linking Time-Varying Symptomatology and Intensity of Infectiousness to Patterns of Norovirus Transmission

**DOI:** 10.1371/journal.pone.0068413

**Published:** 2013-07-24

**Authors:** Jonathan L. Zelner, Benjamin A. Lopman, Aron J. Hall, Sebastien Ballesteros, Bryan T. Grenfell

**Affiliations:** 1 Department of Ecology and Evolutionary Biology, Princeton University, Princeton, New Jersey, United States of America; 2 Epidemiology Branch, Division of Viral Diseases, National Center for Immunization and Respiratory Diseases, Centers for Disease Control and Prevention, Atlanta, Georgia, United States of America; 3 Fogarty International Center, National Institutes of Health, Bethesda, Maryland, United States of America; University of Hong Kong, Hong Kong

## Abstract

**Background:**

Norovirus (NoV) transmission may be impacted by changes in symptom intensity. Sudden onset of vomiting, which may cause an initial period of hyper-infectiousness, often marks the beginning of symptoms. This is often followed by: a 1–3 day period of milder symptoms, environmental contamination following vomiting, and post-symptomatic shedding that may result in transmission at progressively lower rates. Existing models have not included time-varying infectiousness, though representing these features could add utility to models of NoV transmission.

**Methods:**

We address this by comparing the fit of three models (Models 1–3) of NoV infection to household transmission data from a 2009 point-source outbreak of GII.12 norovirus in North Carolina. Model 1 is an SEIR compartmental model, modified to allow Gamma-distributed sojourn times in the latent and infectious classes, where symptomatic cases are uniformly infectious over time. Model 2 assumes infectiousness decays exponentially as a function of time since onset, while Model 3 is discontinuous, with a spike concentrating 50% of transmissibility at onset. We use Bayesian data augmentation techniques to estimate transmission parameters for each model, and compare their goodness of fit using qualitative and quantitative model comparison. We also assess the robustness of our findings to asymptomatic infections.

**Results:**

We find that Model 3 (initial spike in shedding) best explains the household transmission data, using both quantitative and qualitative model comparisons. We also show that these results are robust to the presence of asymptomatic infections.

**Conclusions:**

Explicitly representing explosive NoV infectiousness at onset should be considered when developing models and interventions to interrupt and prevent outbreaks of norovirus in the community. The methods presented here are generally applicable to the transmission of pathogens that exhibit large variation in transmissibility over an infection.

## Introduction

A recent spike in the number and severity of Norovirus (NoV) outbreaks worldwide [1] has underscored the rising costs of NoV transmission in both public health and economic terms. Because NoV transmission is often so explosive that a small number of isolated cases may touch off a large outbreak (e.g. [2]–[5]) the development of effective interventions necessitates an understanding of how each stage of infection in individual cases impacts outbreak transmission.

NoV illness is characterized by multiple phases of symptomatology: an initial period of explosive vomiting characteristic of illness onset, followed by 1–3 days of less severe symptoms [6]. Environmental contamination resulting from episodes of vomiting and diarrhea [2], [4], [5], [7] and post-symptomatic shedding that may persist for days or weeks after symptoms have resolved [8] have also been implicated in transmission.

Representing variable infectiousness over the course of a single infection has been shown to be important for understanding the transmission dynamics of other pathogens, such as HIV [3], [9]. However, despite documented variation in NoV symptom intensity over time, dynamic models of norovirus transmission have typically represented infectiousness as homogeneous over the life of a typical infection (e.g. [7]), or have focused on discrete events such as vomiting within a classroom [10] or the structure of contact networks [11] rather than changes in symptom intensity over the infectious period of a typical case. In this paper, we build upon earlier work demonstrating the importance of vomiting in norovirus transmission to consider the impact of including time-varying intensity of infectiousness on the qualitative and quantitative fit of several models of household-level norovirus transmission. The methods presented here are also generally applicable to the outbreak transmission of other enteric and respiratory pathogens, where variation in symptom-severity across individuals and over time can impact transmission dynamics.

**Figure 1:**
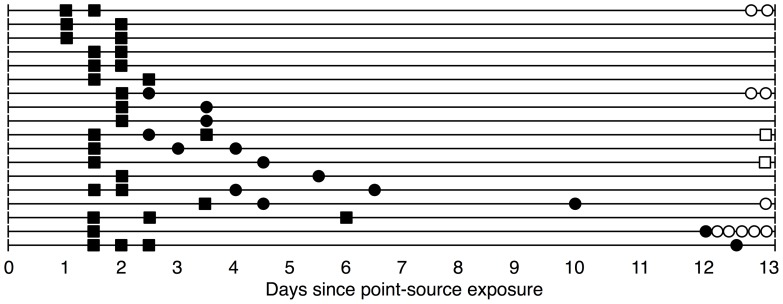
Observations for 18 households with non-index cases. The figure illustrates the time course of infection in the 18 households in which there was a non-index case who became ill after the onset of symptoms in the index case. Filled boxes indicate an individual who dined at the point-source and became ill. Filled circles indicate individuals who became ill and did not dine at the point-source. Hollow boxes and circles along the right margin indicate the number of individuals in the household who did and did not dine at the point source and did not become ill, respectively. The additional 52 households in the analysis with no secondary cases are not pictured.

**Figure 2:**
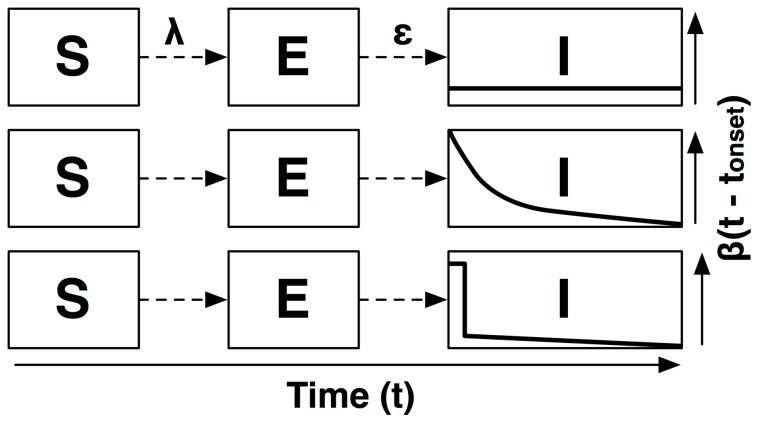
Illustration of model structure for Models 1, 2 & 3. The figure illustrates the change in infectiousness over time, for Model 1 (SEIR; top), Model 2 (Exponential decay; middle) and Model 3 (Burst; bottom). 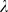 is the force of infection, i.e. the rate at which susceptible individuals are recruited to the latent class, *E*, and 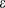 is the mean rate at which infected individuals progress to infectiousness. The asymptomatic class, *A*, and recovered class, *R*, are omitted from the figure for visual clarity.

We fit three models (Models 1–3), each with a different representation of the infectious period, to household transmission data collected subsequent to a 2009 point-source outbreak of GII.12 norovirus in North Carolina [12] and compare the results (for a graphical depiction of the household data, see Figure 1). We begin with a standard compartmental model, denoted Model 1, which allows for heterogeneous infectious period duration but assumes homogeneous infectiousness over time. The first alternative model, Model 2, assumes exponentially decaying infectiousness after onset, while the final model, denoted Model 3, allows for a spike in infectiousness at onset followed by a sharp drop-off in infectiousness 12 hours after the onset of symptoms. For a graphical depiction of these models, see Figure 2.

## Methods

### Data source

In December 2009, more than 200 individuals were sickened by a GII.12 norovirus outbreak caused by contaminated oysters served at a North Carolina restaurant. The particular GII.12 strain implicated in this outbreak is estimated to have caused 16% of reported NoV outbreaks in the United States in 2009–10 [13]. Of the 177 individuals who met the case definition for NoV infection in this outbreak, 85% reported vomiting at some point during their infection [12]. Household transmission subsequent to infection of a household member exposed at the restaurant was assessed via a phone survey. We designate the first individual in a household who exhibited symptoms after dining at the restaurant as the household *index case*. Household contacts of the index case reported whether they dined at the point source and whether they became ill in the 14 days after illness onset in the household index case. Individuals who dined at the point-source but had symptom onset after the index case are denoted as *exposed non-index cases*. Individuals who were not exposed to the point-source but became ill during the observation period are referred to as *secondary cases*. Those who were not observed to be ill are referred to as *non-cases*. For those individuals who reported becoming ill, the approximate time of illness onset (within 12 hours) was obtained.

Because this work was determined by CDC human subjects review to be under the auspices of public health response, the protocol and consent procedure were not formally reviewed by an IRB, though standard practices of verbal consent and confidentiality were followed. The data were collected as part of a phone survey, so it was not possible to obtain written consent. Respondents were assured that all survey questions were voluntary and confidential. Verbal informed consent was requested and documented on the survey instrument at the time of the interview.

Figure 1 illustrates the times of illness onset in the 18 households from this outbreak that had secondary cases. A detailed epidemiological analysis of this outbreak has been presented elsewhere [12].

Because index cases were infected at a point-source event rather than during a large community outbreak that continued throughout the period assessed by the phone survey, we are able to isolate the likely source of exposure to other members of the household. In essence, our household-level transmission data provide multiple independent realizations of the stochastic transmission process, since we observed 70 exposed households, each with a distinct index case. This allows us to examine how time-varying intensity of infectiousness impacts stochastic variability in transmission [7], [8].

### Transmission models

For comparison with our alternative models, we first fit an SEIR compartmental model [14] modified to allow for Gamma-distributed sojourn times in the latent (E) and infectious (I) classes (Model 1), to the outbreak data. In Model 1, infectiousness per unit of time does not change as the infectious period progresses, but infections may be of variable duration. We then develop two alternate models with time-varying infectiousness. The first, Model 2, represents the change in infectiousness as exponentially decaying over time. The other alternative model, Model 3, begins with a sharp spike in infectiousness at onset, dropping off after the first 12 hours of infection. This represents discontinuity in infectiousness at the conclusion of the profuse vomiting that often marks onset. Figure 2 illustrates differences in model structure for Models 1, 2 & 3. Table 1 defines parameters used in Models 1–3.

**Table 1:** Parameters and definitions.

Model	Parameter	Definition	Value	Source
**All Models**	*φ_PS_*	Probability of infection at point source	–	EST
	*ζ*	Relative infectiousness of asymptomatics	0.05	See text
	*ρ*	Proportion of cases asymptomatic	[0.0, 0.4]	Atmar et al. 2006
	*ε*	Mean duration of latency	1 day	See text
	*ε_S_*	Shape parameter of latent period distribution	4	See text
**Model 1**	*β*	Daily symptomatic transmission rate	–	EST
	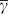	Mean duration of symptomatic infectiousness	–	EST
	*γ_S_*	Shape par. of infectious period duration	–	EST
**Model 2**	*φ_2_*	Total infectiousness	–	EST
	*1/η_2_*	Mean day of infectivity profile	–	EST
**Model 3**	*φ_3_*	Total infectiousness	–	EST
	*1/η_3_*	Mean day of post-onset infectivity	–	EST
	*τ*	Proportion of infectiousness at onset	–	See text

The table lists parameters used in each model as well as fixed values and ranges of parameters assumed or estimated separately from the current analysis. Entries marked ‘EST’ indicate parameters estimated in the analysis. Model-specific parameters are denoted by a subscript.

We then compare the ability of each model to reproduce characteristic features of the outbreak data. This allows us to assess whether a model with time-varying infectiousness may provide a more comprehensive explanation of qualitative features of NoV transmission than standard approaches. Using a quantitative Bayesian model comparison technique, we also compare the two models of time-varying infectiousness to understand if one of these representations may better explain the data.

Because asymptomatic norovirus infection is common, accounting for 15–40% of all norovirus infections [15], we also explore the sensitivity of Models 2 & 3 to the presence of asymptomatic infections. Although asymptomatic cases are unlikely to be as infectious as symptomatic ones [16], they still may impact transmission. Understanding these dynamics is particularly important for quantifying the role of post-symptomatic shedding and persistence of the virus in the environment. Specifically, long serial intervals between symptomatic cases in a household could be explained by either the presence of an asymptomatic case bridging two symptomatic cases, or a long period of environmental contamination or post-symptomatic shedding. For clarity, when discussing the implementation of transmission models in the text, we focus primarily on transmission to and from symptomatic cases. For more detail on the implementation models including asymptomatic cases, see File S1.

### Model 1: SEIR compartmental model

Because the outbreak data are reported in twelve-hour intervals, we use a discrete-time model where each model step represents a 12-hour period. We designate the time *t = 0* to be when the first individual in the household is exposed to the point source. When *t* = 0, we assume that all individuals in the household are in the susceptible state, *S*. The state variable *S(t)* represents the number of susceptible individuals in the household at time *t*. We denote 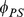 to be the probability that individuals who dined at the point-source were infected there.

Upon infection, individuals enter the latent state (E). The mean duration of latency is estimated from the outbreak data to be 1 day. We represent the latent period with a Gamma distribution with mean 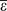 = 1 day, and shape parameter 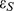 = 4. A shape parameter of 4 represents moderate variability in latency, with 95% of latent periods in the range from 12–48 hours, consistent with data reported from the outbreak [12] and other clinical and outbreak studies [17], [18]. Sensitivity analysis in which the mean duration of latency was increased to 1.5 days did not show any difference in results.

After latency, individuals progress to the symptomatic (I) or asymptomatic (A) phase of infection. We assume that all individuals in the household are equally susceptible to infection, regardless of age, sex or household configuration. Infected individuals will have a symptomatic infection with probability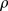 and an asymptomatic infection with probability 1-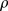. Asymptomatic infections have a fixed proportion, 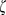, of the infectiousness of symptomatic individuals. When fitting models to the outbreak data, we fix 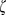 = 0.10, as only qualitative estimates of the infectivity of asymptomatic cases are available (e.g., [8], [16]) and these indicate that they are much less infectious on a per-contact basis than symptomatic cases. We then examine the sensitivity of the model to variability in 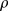, as the proportion of cases that are fully asymptomatic is not well understood.

After latency, individuals enter either the symptomatic infectious period (*I*) with probability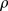, or the asymptomatic infectious period (*A*) with probability 1-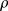. In our model, household NoV transmission is assumed to be density dependent, i.e. each symptomatic individual transmits to each of her susceptible contacts at rate 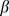 regardless of household size. Infectious period duration is modeled by a Gamma distribution, with parameters 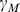
(mean duration), and 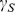 (shape parameter) to be estimated from the data. For 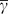 and 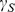
we use Uniform prior distributions on the range (0,100]. For 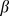 we use a Uniform prior distribution on the range (0, 10]. We can write the force of infection on a susceptible individual from the household transmission as 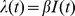, where *I(t)* is the number of symptomatic individuals in the household at time *t*.

### Model 2: Exponential decay of infectiousness

Model 2 introduces smooth variation in infectiousness over time. In this model, the infectiousness of a case decays exponentially as a function of time elapsed since the onset of symptoms. We use a discretized exponential distribution with mean
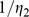
days, denoted 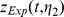, where 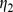 is the rate parameter of the distribution, to represent the proportion of an individual's infectiousness occurring at each 12-hour interval after onset, denoted by *t*_onset_. An exponential distribution is a natural choice to represent infectivity over the symptomatic period, because its mode is at zero [19], i.e., the time of symptom onset. This guarantees that the largest amount of infectiousness occurs with the onset of symptoms. It is also a parsimonious representation of the change in infectiousness over time, as the distribution has only a single parameter controlling the rate of decay. So, 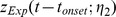
is the proportion of the individual's infectivity that occurs during period *t*. This is referred to as the *infectivity profile* of the case [20], [21]. The total expected transmission rate over the entire infection is denoted 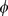, and the rate of transmission from a case to a susceptible individual at a given time, *t*, is 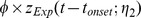.

We denote 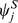
to be the time of symptom onset. The force of infection on a susceptible individual *j* from an infectious case, *i,* at time *t* can then be calculated as follows:
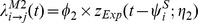



### Model 3: Burst of infectiousness followed by exponential decay

In Model 3, the infectious period consists of two phases: 1) a 12-hour burst of infectivity starting with the onset of symptoms, followed by 2) a period of declining infectivity modeled by an exponential distribution with mean 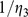, as in Model 2. The burst in infectiousness is assumed to last for only the initial 12 hours of the infectious period, as the majority of vomiting during naturally-infected norovirus cases has been observed to occur in the first 24 hours after infection [15]. In addition, the onset of vomiting is often sudden and explosive, so contact-limiting behaviors that minimize transmission even in the presence of vomiting are likely to be implemented in the period immediately following the initial vomiting event. Because the sudden onset of vomiting is such a characteristic feature of symptomatic NoV infection, Model 3 is meant to represent the risk associated with a typical case, although individual cases may deviate from this pattern. For example, variation in the location, magnitude, and number of vomiting events associated with an individual case may contribute to between-individual heterogeneity.

Model 3 introduces a new parameter, 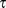, which defines the proportion of a case's infectiousness that occurs during the 12 hour burst at onset, with the remaining 1-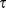 occurring afterwards. We fix 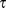 = 0.5, so that 50% of the infectiousness occurs during the burst immediately after onset, with the remaining half spread over a period of exponentially decaying infectivity. This ensures that comparisons with Model 3 highlight the differences between a model assuming that vomiting characteristic of illness onset concentrates a disproportionate amount of infectiousness at the period immediately following the start of symptoms, and those that do not. We denote the rate of transmission over the life of the infection as 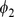, and define the force of infection from a symptomatic individual as a function of time as follows:
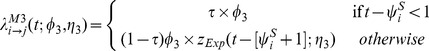



### Data model

We fit Models 1–3 to the outbreak data using a Bayesian data augmentation (DA) approach [20]. These methods allow us to model outbreak data that are partially observed. Because we only observe the time of illness onset but not infection or recovery it is not possible to directly calculate the likelihood. DA methods allow us to use Markov-Chain Monte Carlo (MCMC) tools to sample from the posterior distribution of model parameters and unobserved events (infection, recovery) in the individual's infection history. Sampling complete sequences of events makes the likelihood function tractable and allows us to estimate model parameters. This approach has previously been used to model norovirus outbreaks [7] as well as transmission of influenza [21], [22] pneumococcus [23], [24], and foot and mouth disease [25], among other pathogens. In Model 1, there are two unobserved events corresponding to each non-index case: the infection time and recovery time. In Models 2 & 3, there is only one latent event per case: the infection time.

When fitting parameters for the mean day of the infectivity profile for Models 2 & 3 (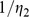,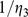), we use a uniform prior on the interval from 1–5 days. This was done to constrain the sampler to plausible durations of infectiousness and to guard against overfitting of the models to households with longer serial intervals between cases. For models 2 & 3, a Uniform prior on the range (0, 10] was used for the parameter 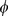. When estimating parameter values, we use the mean of the parameter's marginal posterior distribution. We checked these values against the corresponding posterior median and found that our results are not sensitive to this choice.

To understand the contribution of fully asymptomatic infections, i.e. those that enter the asymptomatic infectious phase, *A*, immediately after the end of the latent period, *E*, we employ two reversible jump MCMC steps (see, e.g.[23], [24]) that allow us to add and remove asymptomatic infections from the histories of non-cases in the outbreak dataset. Because asymptomatic infections are likely to be much less infectious than symptomatic ones, our motivation for including them is to understand their impact on estimates of the symptomatic transmission rate. It is not feasible to estimate transmission parameters for asymptomatic infections from our data because they are unlikely to emit a strong signal of infectiousness. Consequently, we make some assumptions about the nature of asymptomatic infections, specifically: 1) that they are only 10% as infectious as symptomatic infections and 2) that the mean of the infectivity profile is at 5 days. For a detailed explanation of the application of these methods to our data, see File S1.

All models presented here were implemented using *Python 2.7*. Analysis of posterior distributions was performed using the *coda* package in *R 2.15*. Code used in this analysis is available from the authors upon request.

### Model comparison

We compare the quality of model fit for Models 1, 2 & 3 based on qualitative fit to the data based on descriptive statistics. We perform Bayesian model selection using Bayes factors [21] to compare the time-varying infectiousness models (2 & 3).

### Qualitative model comparison

To make qualitative comparisons, we fix the parameters for each model at the posterior mean values estimated from the outbreak data and generate 10^4^ sample outbreaks using the household sizes and point-source exposure patterns from the outbreak data. We then compare features of these sampled outbreaks to the household outbreak data via several descriptive statistics. These include: 1) the average number of secondary cases in households with at least one secondary case, 2) the average serial interval between household cases, 3) the average time from onset in the first household case to onset in the last household case in those households with secondary cases, 4) the probability of zero observed secondary cases within the household, i.e. that the index fails to generate any secondary cases, and 5) the probability of recrudescence of a household outbreak, defined here as the probability of observing a serial interval of ≥4 days between cases in a household.

### Quantitative model comparison

We compare the relative strength of evidence supporting these models using Bayes factors. Bayes factors facilitate the comparison of models with differing structure. However, the large number of unobserved recovery times in Model 1, each represented by a hidden parameter, makes meaningful comparison of Model 1 with Models 2 & 3 with Bayes factors infeasible. This is because Bayes factors naturally penalize additional model structure, effectively guaranteeing that a model with fewer latent parameters (i.e., Models 2 &3) would be preferred over one with many more. So, we limit this aspect of the analysis to the two models with varying infectiousness over the symptomatic period. The Bayes factor, K_32_, that we use to compare the models M_2_ & M_3_ is the ratio of the posterior densities of each *model*, given the data, i.e. 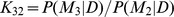 (see [21] & File S1).

Because we repeat this analysis for varying rates of asymptomatic prevalence, we denote 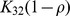 to be the Bayes factor comparing Model 3 to Model 2, for a specific level of asymptomatic prevalence, 1-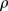. We calculated Bayes factors for each 10% increment in asymptomatic prevalence from 0% to 40%. This allows us to examine the strength of evidence in favor of each model under different assumptions about the prevalence of asymptomatic infections. To interpret these Bayes factors, we use the Jeffreys scale [26], which is a subjective guide to the strength of the evidence supporting a given hypothesis.

In order to perform this portion of the analysis, we employed a reversible-jump MCMC sampling step that allowed the sampler to jump between Models 2 and 3 within a single MCMC run. This step is similar to one employed by O'Neill & Marks [10] in their analysis of NoV transmission in a school outbreak.

When interpreting the results of these model comparisons, it is important to note that Model 3 represents a stylized scenario in which 50% of infectiousness is concentrated at onset. This means that comparisons between Model 2 and 3 are necessarily heuristic in terms of their ability to assess the explanatory power of a model that allows excess infectivity at onset versus one that does not. So, for example, if Model 3 is preferred over Model 2, this would indicate that a model with 50% of infectiousness concentrated at onset is preferred to one in which there is an exponential decay of infectiousness beginning at onset. But it should not be taken as evidence that a model with a spike in infectiousness at onset is preferred in all cases, regardless of the height of this spike.

## Results

### Parameter estimates

Table 2 lists parameter estimates for Models 1–3 with only symptomatic infections. For estimates over varying levels of asymptomatic prevalence, see Tables S1 & S2 in File S1. For descriptive characteristics of the household outbreaks, see Table 3.

**Table 2:** Parameter estimates for Models 1–3.

Model	Para meter	Model 1		Model 2		Model 3	
		Est	95% CI	Est	95% CI	Est	95% CI
**All**	*φ_PS_*	0.55	(0.38, 0.71)	0.55	(0.38, 0.71)	0.54	(0.38, 0.71)
**Time Varying**	*φ*	–	–	0.13	(0.07, 0.21)	0.14	(0.08, 0.22)
	*η*	–	–	2.78	(1.55, 4.87)	3.89	(2.20, 4.95)
**Compart mental**	*β*	0.05	(0.02, 0.10)	–	–	–	–
	*γ*	2.98	(1.22, 4.79)	–	–	–	–
	*γ_S_*	1.04	(0.07, 3.63)	–	–	–	–

**Table 3:** Descriptive characteristics of household outbreak data compared to 10?4 simulations from fitted Models 1–3.

	Data		Model 1		Model 2		Model 3	
Descriptive Statistic	Value	Range	Value	5^th^–95^th^ Quantile	Value	5^th^–95^th^ Quantile	Value	5^th^–95^th^ Quantile
Avg#secondary cases (in hh w/1+ cases)	1.3	(1.0, 4.0)	1.2	(1.0, 3.0)	1.3	(1.0, 3.0)	1.3	(1.0, 3.0)
Avg household outbreak****duration (days)	3.1	(0.5, 10.0)	2.8	(0.5, 10.0)	3.4	(0.5, 11.0)	2.9	(0.5, 10.0)
Avg serial interval (days)	2.3	(0.5, 8.0)	2.6	(0.5, 8.0)	2.7	(0.6, 9.0)	2.3	(0.5, 9.5)
Probability no****secondary cases	0.62	–	0.70	–	0.63	–	0.64	–
Recrudescence****probability	0.17	–	0.22	–	0.29	–	0.21	–

**Figure 3:**
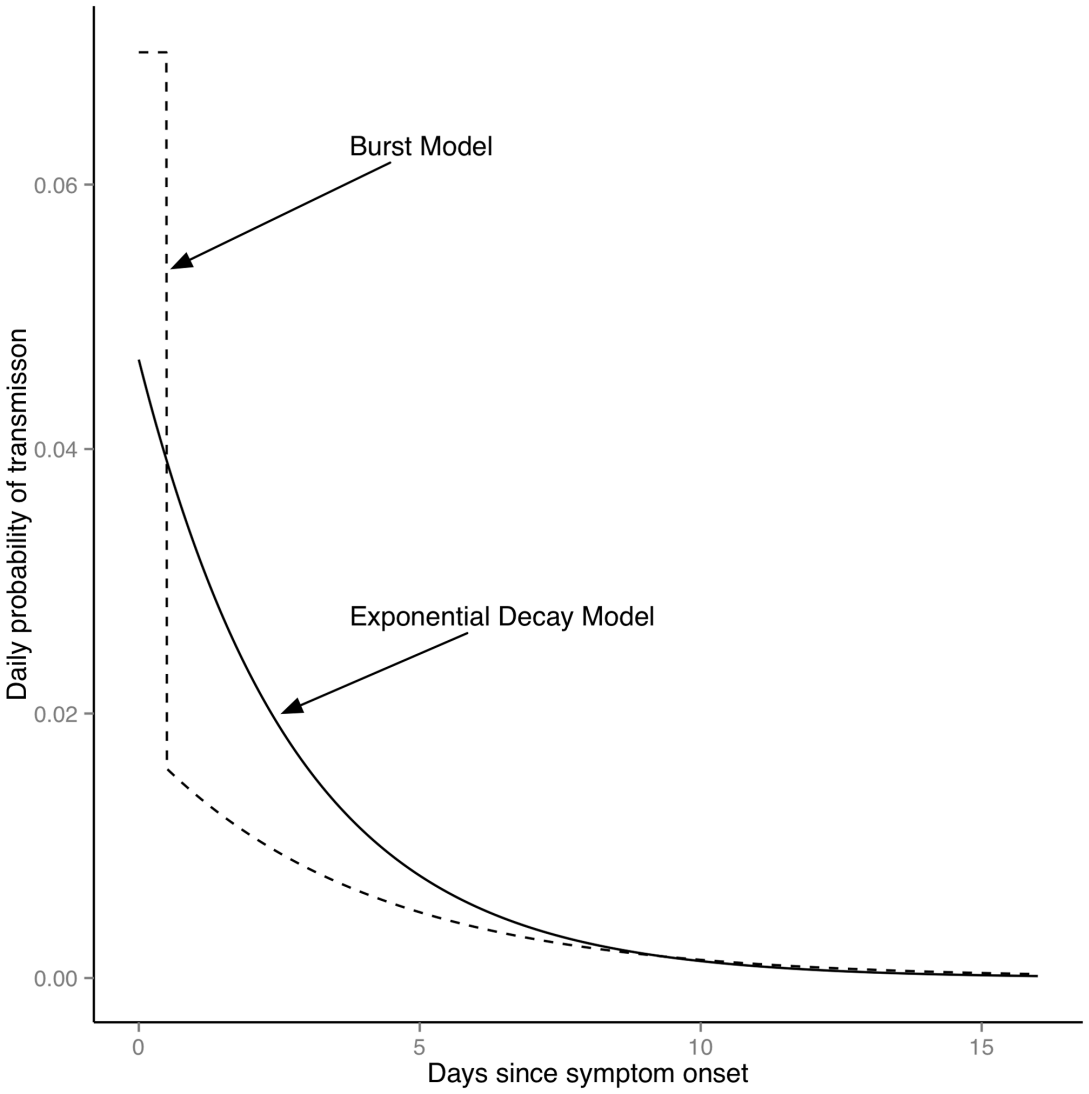
Fitted infectivity profiles for Models 2 & 3. The figure shows infectiousness as a function of time since symptom onset for the estimated values of the exponential decay model (Model 2; solid line) and burst model (Model 3; dashed line).

Across all three models, estimates of the probability of infection at the point source are similar, with a 55% probability of infection for non-index cases exposed to the point source (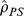 =  0.55, 95% CI  = 0.38, 0.71. Estimates of a secondary attack rate of approximately 15% are also consistent across models. To test the validity of the assumption in Model 3 that 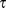 =  0.5, i.e. that 50% of infectiousness occurs during the burst at onset and 50% occurs afterwards, we re-fit Model 3 while allowing 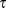 to vary. We found that the estimated value was close to the assumed value (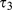 =  0.46, 95% CI: 0.19, 0.75), while other parameter estimates listed in Table 2 were unchanged. Sensitivity analysis using Bayes factors was also performed in which the duration of the burst at onset was varied from 12 to 24 hours, and both burst durations were supported equally by the data with no impact on estimated parameter values. Figure 3 illustrates the fitted infectivity profiles for Models 2 and 3.

### Model comparison

Table 3 presents descriptive features of the outbreak data and corresponding measures of 10^4^ of simulated outbreaks from Models 1–3. The first column of Table 3 lists mean values from the data and the ranges of these values. In Columns 2–4, is the mean value from the simulations and values spanning the range from the 5^th^ to the 95^th^ quantile of the simulated distribution of the outcome. We find that all models reproduce mean values and variability in the outbreak data for 1) the number of secondary cases, 2) duration of serial intervals between cases, and 3) average household outbreak duration. The models differ in their ability to reproduce recrudescence and the proportion of households with no secondary cases. The probability of recrudescence for Model 1 (0.22) and Model 3 (0.21) are closest to the data (0.17), while Model 2 overestimates this value by a larger margin than the other two (0.29). Simulations from Models 2 & 3 generate values for the probability of observing no secondary cases (0.63 & 0.64, respectively) that are closer to the data (0.62) than Model 1 (0.70). Overall, Model 3 generates values closest to the outbreak data along those qualitative dimensions where there are differences between the candidate models.

In our quantitative comparison of models 2 and 3, we find that in a model with only symptomatic infections, support for Model 3 is strong (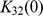 = 25). As asymptomatic prevalence increases from 10% to 40%, the strength of evidence in favor of Model 3 decreases, but remains strong (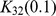 = 16, 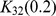 = 14, 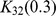 = 14, 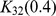 = 16). This indicates that the explanatory power of Model 3 is not sensitive to the level of asymptomatic prevalence. Sensitivity analysis in which the infectiousness of an asymptomatic case relative to a symptomatic case was varied from 5% to 15% did not impact these results. Tables S1 & S2 in File S1 show parameter estimates for models with varying levels of asymptomatic prevalence. Tables S3 & S4 in File S1 show the robustness of the fitting procedure to inclusion of asymptomatic infections.

## Discussion

These results suggest that models including time-varying infectiousness may better capture observed person-to-person norovirus transmission dynamics than approaches assuming uniform intensity of infectiousness over time. Allowing for changes in infectiousness that reflect characteristic patterns of norovirus illness can increase our ability to explain observed outbreak patterns and re-create qualitative features of these outbreaks. In particular, Models 2 & 3 were better able than Model 1 to reproduce the proportion of household outbreaks not resulting in secondary cases. Model 3 was also able to capture the probability of recrudescence in household outbreaks, potentially because the infectiousness remaining after the burst at onset is more evenly distributed over the infectious period than in Model 2. A particular strength of an approach allowing for symptom intensity to vary with time is that the roles of waning symptomatology and post-symptomatic shedding can be explored without adding model complexity, i.e. additional infectious classes. Because it eliminates latent state variables in the infectious period, this framework also facilitates straightforward model comparison.

Quantitative comparisons between Models 2 and 3 suggest that Model 3 provides a more comprehensive picture of the outbreak data than the other models presented here. This also holds across a range of plausible asymptomatic prevalence rates. These results are qualified, however, by the relatively small size of this outbreak and should be verified against datasets with a higher density of cases. It is important to note, however, that our data actually represent 70 independent replications of the household transmission process. In addition, the use of a dataset where an individual's outcomes are directly linked to her exposure is likely to decrease error in estimation relative to approaches in which only the aggregate force of infection and population-level incidence are considered, (see e.g. [27]). These findings also echo results from other modeling studies which have found that asymptomatic infection is unlikely to play a major role in person-to-person transmission during an outbreak [16].

Although all three models are able to explain key features of the data, the qualitative fit of model 3 is the strongest of those considered here. As compared to models 1 & 2, it is able to capture both patterns of within-household transmission as well as the probability the index case will fail to transmit to any household members. Our findings are, however, limited by two factors.

First, the fact that our dataset consists primarily of self-reported illness onset times may introduce some error with respect to the actual time of infectiousness onset. Second, to provide a contrast to models 1 & 2, in model 3 the proportion of a case's infectivity at onset is fixed at 50%. This means that our model comparison results need to be interpreted as a contrast between one in which 50% of the infectiousness occurs at onset with smooth variation thereafter to one in in which infectiousness at onset is tied smoothly to variation afterwards, rather than a general comparison between a model with a spike at onset and one in which there is no such spike.

Consequently, although our findings suggest that it is important to account for increased infectiousness at onset, they should be verified and expanded using outbreak datasets with more cases and larger contact networks. Future analysis should also address variation in infectivity profiles by age, as this is likely to influence transmission. In addition, data including laboratory testing confirming symptomatic infection and identifying asymptomatic cases is necessary to verify the robustness of these results to asymptomatic transmission.

In model 3, we also assume that the 12-hour duration of the burst of infectiousness following onset is similar to the period of vomiting reported from a cohort study of norovirus infections in the community [15]. Clinical challenge studies, e.g. [8], have shown longer periods of vomiting of up to several days. But this may result from dosage with quantities of norovirus much greater than likely to be encountered in the context of a real-world outbreak. Given the critical role of vomiting in norovirus transmission [3], [10], further study is necessary to understand the distribution of the duration and intensity of vomiting in the context of real-world outbreaks and the role of contact-limiting behavior in transmission.

The methods discussed here may be extended to include the mechanisms driving the shape of infectiousness over time. For example, for HIV and other STIs, infectiousness over time may be modeled as a function of individual-level covariates, such as changes in risk behavior. Models including time-variation in the influence that individuals have on each other might also be usefully extended to studies of the diffusion of behavioral risks for chronic illness, e.g. obesity [28], where compartmental modeling approaches may result in an awkward discretization of changes in social behavior over time.

Our results underscore the idea that public health interventions need to focus on both the acute phase of infection as well the environmental contamination and post-symptomatic infectiousness that characterize norovirus outbreaks. Onset of norovirus gastroenteritis is often abrupt, with no prodrome, so public vomiting events are common. Preventing such events from occurring may not be possible, but our results demonstrate the importance of rapidly responding to such occurrences, as well as other opportunities for transmission in the initial phase of illness. Future work should test this framework and its implications for intervention in the context of community and institutional outbreaks, where issues of sanitation are most acute.

## Supporting Information

File S1 **Combined supplementary materials file.**
(PDF)
