# Supplementary material for: Linking Time-Varying Symptomatology and Intensity of Infectiousness to Patterns of Norovirus Transmission
Source: PLoS One. 2013 Jul 24;8(7):e68413. doi: 10.1371/journal.pone.0068413 (PMC3722229; doi:10.1371/journal.pone.0068413)
Supplement: File S1 — Combined supplementary materials file. (PDF) [file pone.0068413.s001.pdf]

Supplementary Materials

S1. Data Augmentation Methods

In this section we outline the methods used to fit each transmission models to the observed household transmission data. When describing the fitting procedure, we adapt the notation of Cauchemez and Ferguson [1]. For clarity, we first define the likelihood as though the times of all events, i.e. infection times and onset times for both symptomatic and asymptomatic cases are known. We then outline a Markov-Chain Monte Carlo (MCMC) algorithm used to systematically explore the space of unobserved infection times (and onset times for asymptomatic infections) necessary to calculate the likelihood.

Augmented Data Likelihood

Individuals within a household are indexed by i; for each individual $i = 1 \dots N$, we define $y_i^S = 1$ if the individual is a non-index case with a symptomatic infection between time 0, the time of exposure of the household index case, and T, the end of the observation period. We define $y_i^A = 1$ if the individual had an asymptomatic infection on the interval [0, T]. We denote the time of infection for each non-index, symptomatic case as t_j^S and denote this time as t_j^A for each asymptomatic case. For economy of notation, we will refer to asymptomatic and symptomatic infection times together as t_j^*.

By convention, individuals who are not infected during the observation period are assigned $t_j^* = T + 1$, where T is the duration of the observation period. Finally, $f(x; \epsilon^{-1}, \epsilon_S)$ is the density of the incubation period duration, a Gamma distribution parameterized in terms of its mean duration in days (ϵ^{-1}) and shape parameter (ϵ_S).

We can now calculate the contribution that symptomatic and asymptomatic cases, as well as non-cases, make to the likelihood.

First, for symptomatic cases:

$$
\begin{aligned}
&P(y_j^S; t_j^S = t) \\
&= f(\psi_j^S - t_j^S; 1/\epsilon, \epsilon_S) \\
&\times \left\{ 1 - exp\left(-(1-\rho) \times \sum_{\{i:y_i^*=1; t_i^*<t\}} \lambda_{i\to j}(t|\theta) \right) \right\} \\
&\times exp\left(\sum_{\{i:y_i^*=1; t_i^*<t\}} \sum_{d=t_i^*}^{t-1} \lambda_{i\to j}(d|\theta) \right)
\end{aligned}
$$

And then for asymptomatic cases:

$$
\begin{aligned}
&P(y_j^A; t_j^A = t) \\
&= f(\psi_j^A - t_j^A; 1/\epsilon, \epsilon_S) \\
&\times \left\{ 1 - exp\left(-\rho \times \sum_{\{i:y_i^*=1; t_i^*<t\}} \lambda_{i\to j}(t|\theta) \right) \right\} \\
&\times exp\left(- \sum_{\{i:y_i^*=1; t_i^*<t\}} \sum_{d=t_i^*}^{t-1} \lambda_{i\to j}(d|\theta) \right)
\end{aligned}
$$

Finally, we account for the contribution of non-cases to the likelihood:

$$
P(y_j^S = 0, y_j^A = 0) = exp\left(- \sum_{\{i:y_i^*=1\}} \sum_{d=t_i}^{t-1} \lambda_{i\to j}(d|\theta) \right)
$$

The product of the contributions of all cases and non-cases in a household h is the augmented data likelihood, $P(D_h, Y_h|\theta)$, for that household:

$$
P(D_h, Y_h|\theta) = \prod_{\{i:y_{i,h}^S=1\}} P(y_{i,h}^S) \times \prod_{\{i:y_{i,h}^A=1\}} P(y_{i,h}^A) \times \prod_{\{i:y_{i,h}^*=0\}} P(y_{i,h}^* = 0)
$$

Because we assume that the transmission process in each household following exposure at the point source is independent, we can then calculate the sampling probability for the entire dataset as $P(D, Y\ |\theta) = \sum_{i=1}^{N_H} P(D_i, Y_i|\theta)$.

S2. Markov-Chain Monte Carlo (MCMC) Sampling Algorithm

In this section, we outline the MCMC algorithm used to sample the joint posterior distribution of event times and transmission parameters.

A. Adjusting Infection Times

Because our household transmission data are reported in terms of incidence, exact infection times for non-index cases are unobserved. To sample these missing data, we use a Gibbs sampling [2] step in which a case's infection time is sampled directly from the joint distribution of susceptible period durations and incubation period durations, conditional on the time of illness onset.

To do this, we first calculate the probability of 1) infection on each day prior to the onset of symptoms, s_i (where $s_i \in S_t$, the set of all other symptom onset times in the household), and 2) the probability of an incubation period duration equal to $s_i - t$:

$$
\begin{aligned}
&P(t_j^S = t | s_j, S_t) \\
&= f(s_j|t) \left\{ 1 - exp \left(-(1-\rho) \sum_{i:t_i<t} \lambda_{i \to j}(t|s_i) \right) \right\} \\
&\times exp \left(- \sum_{i:t_i<t} \sum_{d=t_i}^{t-1} \lambda_{i \to j}(d|\theta) \right)
\end{aligned}
$$

We then normalize this distribution of potential infection times by conditioning on the total probability that the infection time occurred on the interval $[0, s_i\text{-}1]$, i.e. that it occurred before the onset of symptoms:

$$
p_j(t) = \frac{P(s_j, t_j = t | S_t)}{\sum_{u<s_j} P(s_j, t_j = u | S_t)}
$$

And the new infection time of the case is then sampled from p_j. For more detail on this step, see Appendix A of [1].

B. Adjusting Recovery Times

To sample the unobserved recovery times in Model 1, we use a Metropolis-Hastings step in which a new recovery time, r_i' is proposed from the hypothesized distribution of the infectious period duration, $g(r;\ 1/\gamma^t, \gamma_s^t)$, where γ^t is the rate parameter for the recovery period distribution on the current step, t, of the sampling algorithm and γ_s^t is its shape parameter. Because new times are sampled independently of each other, the proposal ratio, π, for this move reduces to:

$$\pi = \frac{g\left(r_i^t; \frac{1}{\gamma^t}, \gamma_t^s\right)}{g\left(r_i'; \frac{1}{\gamma^t}, \gamma_t^s\right)}.$$

C. Reversible-Jump MCMC Moves for Asymptomatic Infections

To explore the role of asymptomatic infections in these household outbreaks, we use a pair of reversible-jump MCMC moves to insert and remove asymptomatic infections from a household outbreak. Because these infections are completely unobserved, we need to sample both the infection and onset times for such infections. We assume that the incubation periods for asymptomatic infections follow the same distribution as symptomatic ones. We also use a Metropolis-Hastings (MH) move, similar to the one in S2, section B above, to adjust infection and onset times for asymptomatic infections. As with the symptomatic cases, the sampling probability of the latent period for asymptomatic cases is included in the augmented data likelihood.

Here we outline the calculation of proposal probabilities for reversible jump MCMC moves for adding and removing asymptomatic infections:

Move 1: Add Asymptomatic Infection. When adding an asymptomatic infection, we first sample a susceptible individual at random from the set of susceptible individuals across all household, S, with probability N_S^{-1}, where N_S is the size of the susceptible population. We then sample a time of infection from the joint distribution of susceptible period lengths and infection times. To do this, we first calculate the sampling probability of each susceptible period/infection time combination, $P\left(t_j^A = t \middle| S_t, A_t\right)$, which is calculated as follows:

$$
\begin{aligned}
&P(t_j^A = t|S_t, A_t) \\
&= \left\{1 - exp\left(-\rho \sum_{i:t_i<t} \lambda_{i\to j}(t|s_i)\right)\right\} \\
&\times exp\left(-\sum_{i:t_i<t}\sum_{d=t_i}^{t-1} \lambda_{i\to j}(d|\theta)\right)
\end{aligned}
$$

And then sample the infection time from the normalized distribution:

$$
p_j^A(t) = \frac{P(t_j^A = t|S_t, A_t)}{\sum_{u<T} P(t_j^A = u|S_t, A_t)}
$$

Finally, we sample the duration of the latent period from its density, $f\left(t'_{j,s}\right)$, as in S2.B, above. The proposal probability of this step is then $N_S^{-1} \times p_j^A\left(t'_j\right) \times f\left(t'_{j,s}\right)$.

Move 2: Remove Asymptomatic Infection. When removing an asymptomatic infection, we sample an individual at random from the population of individuals who

have an asymptomatic infection, A, and remove the infection. Consequently, the proposal probability for this step is N_A^{-1}.

Using the derivation of the proposal probabilities for Steps 1 and 2, we can easily calculate the proposal ratio, π, for each step. For Move 1:

$$\pi = \frac{1/(N_A + 1)}{N_S^{-1} \times p_j^A(t_j') \times f(t_{j,s}')}$$

For Move 2:

$$\pi = \frac{1/(N_S + 1) \times p_j^A(t_j) \times f(t_{j,s}))}{N_A^{-1}}$$

Move 3: Adjust Asymptomatic Infection and Onset Times. In this step, we sample a new infection and onset time for the asymptomatic case, in the same way as when we add a new infection in Move 1. This results in a proposal ratio $\pi = p_j^A(t_j') \times f(t_{j,s}') / p_j^A(t_j) \times f(t_{j,s})$.

S3. Model Comparison with Bayes Factors.

To compute Bayes factors for the comparison of Models 2 & 3, we employed an MCMC sampling step to switch between models of time-varying infectiousness. For example, if we want to switch from Model 2 to Model 3, we set the parameters ϕ_2, η_2 of Model 2 to zero with probability = 1. We then propose new values for the parameters of Model 3 ϕ_3, η_3, from the proposal distributions $f(\phi_3)$ and $g(\eta_3)$. So, the proposal ratio for a move from Model 2 to Model 3 is:

$$\pi_{2\to3} = \frac{f(\phi_2)g(\eta_2)}{f(\phi_3)g(\eta_3)}$$

We can then compute the acceptance probability as discussed above. The index of the accepted model is then recorded, and Bayes factors comparing Models 2 & 3 are calculated using the ratios of the marginal posterior densities for each model.

S4. Supplementary Results

Asymptomatic Infections

Table S1 shows estimated parameter values for Model 2 for asymptomatic prevalence increasing from 10% to 40%. Table S2 shows estimated parameters for Model 3 for asymptomatic prevalence ranging from 10% to 40%. In both cases, the estimate of the overall transmission rate scales linearly with asymptomatic prevalence, suggesting at most a weak effect of asymptomatic cases on household transmission dynamics. These results are similar to findings from [3]. The value for the infectivity of the point-source event, ϕ_{PS} appears to be very sensitive to the inclusion of asymptomatic infections. This is likely because these infections are not anchored to an observed onset time, allowing the model to place them at the time the individual dined at the point-source.

Simulated Data

To verify that the fitting procedures used for models both with and without asymptomatic infectious are accurate, we simulated outbreaks using fitted parameter values for Models 2 & 3 from Tables 2, 3 & 4 in the main text, and re-fit the model to these simulated data. We then repeated this procedure for each level of asymptomatic prevalence from 0-40%. When simulating outbreaks, we retain the size of each household in the analysis, as well as whether each individual dined at the point source. For a full simulated data analysis of Model 1, see [3].

As in the main text, we constrain the search for the mean day of infection when estimating Models 2 & 3. Simulated data results for Model 2 are presented in Table S3, and results for Model 3 are presented in Table S4. For all except one parameter across all levels of asymptomatic prevalence, parameter values used in simulations are included in the estimated 95% credible intervals and, in most cases, are very near to the estimated value. A notable exception is the value of ϕ, the overall transmission rate, for the 30% asymptomatic prevalence level in Model 3. In this case, the estimated value was much larger than the simulated one. This highlights the fact that Monte Carlo error may influence our results, although re-estimation with other simulated datasets (not shown) obtained more accurate results.

	Asymptomatic Prevalence							
	10%		20%		30%		40%	
	**Est**	**95% CI**	**Est**	**95% CI**	**Est**	**95% CI**	**Est**	**95% CI**
Point-source events								
Probability of infection at point-source	0.96	(0.66, 0.99)	0.98	(0.90, 0.99)	0.98	(0.13, 0.38)	0.98	(0.92, 0.99)
Symptomatic Transmission								
Phi (total infection)	0.17	(0.09, 0.27)	0.19	(0.11, 0.32)	0.23	(0.13, 0.38)	0.28	(0.15, 0.48)
Eta (mean of infectivity profile)	3.10	(1.67, 4.81)	3.12	(1.71, 4.82)	3.12	(1.70, 4.81)	3.13	(1.72, 4.82)
Asymptomatic Transmission								
Phi_A (total infection)	0.02	(0.01, 0.03)	0.02	(0.02, 0.03)	0.02	(0.01, 0.04)	0.03	(0.01, 0.05)

Table S1. Transmission parameter estimates for increasing asymptomatic prevalence for Model 2.

	Asymptomatic Prevalence							
	10%		20%		30%		40%	
	**Est**	**95% CI**	**Est**	**95% CI**	**Est**	**95% CI**	**Est**	**95% CI**
Point-source events								
Probability of infection at point-source	0.94	(0.60, 0.99)	0.98	(0.90, 0.99)	0.98	(0.90, 0.99)	0.97	(0.92, 0.99)
Symptomatic Transmission								
Phi (total infection)	0.17	(0.10, 0.28)	0.21	(0.11, 0.33)	0.24	(0.13, 0.39)	0.30	(0.16, 0.50)
Eta (mean of infectivity profile)	3.87	(2.26, 4.95)	3.87	(2.25, 4.95)	3.88	(2.25, 4.95)	3.89	(2.27, 4.95)
Asymptomatic Transmission								
Phi_A (total infection)	0.02	(0.01, 0.03)	0.02	(0.01, 0.03)	0.02	(0.01, 0.04)	0.03	(0.02, 0.05)

Table S2. Transmission parameter estimates for increasing asymptomatic prevalence for Model 3.

	Asymptomatic Prevalence														
	0%			10%			20%			30%			40%		
	**Actual**	**Est**	**95% CI**	**Actual**	**Est**	**95% CI**	**Actual**	**Est**	**95% CI**	**Actual**	**Est**	**95% CI**	**Actual**	**Est**	**95% CI**
Point-source events															
Probability of infection at point-source	0.50	0.45	(0.29, 0.62)	0.95	0.99	(0.99, 1.0)	0.95	0.99	(0.99, 1.0)	0.95	0.99	(0.99, 1.00)	0.95	0.99	(0.99, 1.0)
Symptomatic Transmission															
Phi (total infection)	0.14	0.16	(0.10, 0.24)	0.17	0.14	(0.08, 0.23)	0.22	0.18	(0.10, 0.29)	0.27	0.21	(0.11, 0.34)	0.34	0.31	(0.18, 0.49)
Eta (mean of infectivity profile)	2.50	3.10	(1.78, 4.75)	2.50	3.20	(1.75, 4.83)	2.50	2.74	(1.47, 4.62)	2.50	2.03	(1.07, 3.89)	2.50	2.87	(1.62, 4.64)
Asymptomatic Transmission															
Phi_A (total infection)	0.007	0.02	(0.01, 0.02)	0.0085	0.01	(0.008, 0.02)	0.011	0.02	(0.01, 0.03)	0.0014	0.017	(0.01, 0.03)	0.017	0.03	(0.01, 0.05)

Table S3. Parameter values and estimates for simulated data analysis of Model 2

	Asymptomatic Prevalence														
	0%			10%			20%			30%			40%		
	**Actual**	**Est**	**95% CI**	**Actual**	**Est**	**95% CI**	**Actual**	**Est**	**95% CI**	**Actual**	**Est**	**95% CI**	**Actual**	**Est**	**95% CI**
Point-source events															
Probability of infection at point-source	0.50	0.41	(0.26, 0.56)	0.95	0.99	(0.99, 1.0)	0.95	0.99	(0.98, 1.0)	0.95	0.95	(0.82, 1.0)	0.95	0.99	(0.99, 1.0)
Symptomatic Transmission															
Phi (total infection)	0.14	0.13	(0.07, 0.21)	0.17	0.19	(0.11, 0.29)	0.22	0.25	(0.15, 0.39)	0.27	0.56	(0.35, 0.85)	0.34	0.37	(0.21, 0.62)
Eta (mean of infectivity profile)	2.50	2.30	(0.80, 4.67)	2.50	2.23	(0.90, 4.59)	2.50	2.20	(0.98, 4.43)	2.50	2.05	(0.94, 4.27)	2.50	1.81	(0.67, 4.3)
Asymptomatic Transmission															
Phi_A (total infection)	0.01	0.01	(0.007, 0.02)	0.0085	0.02	(0.01, 0.03)	0.02	0.03	(0.02, 0.04)	0.03	0.05	(0.03, 0.08)	0.03	0.04	(0.02, 0.06)

Table S4. Parameter values and estimates for simulated data analysis of Model 3

Supplementary References

1. Cauchemez S, Ferguson NM (2011) Methods to infer transmission risk factors in complex outbreak data. Journal of the Royal Society Interface. doi:10.1098/rsif.2011.0379.

2. Robert C, Casella G (2004) Monte Carlo Statistical Methods. Springer Verlag.

3. Zelner JL, King AA, Moe CL, Eisenberg JNS (2010) How infections propagate after point-source outbreaks: an analysis of secondary norovirus transmission. Epidemiology 21: 711–718. doi:10.1097/EDE.0b013e3181e5463a.
